# Phylogenetic pattern of SARS-CoV-2 from COVID-19 patients from Bosnia and Herzegovina: Lessons learned to optimize future molecular and epidemiological approaches

**DOI:** 10.17305/bjbms.2020.5381

**Published:** 2021-08

**Authors:** Teufik Goletic, Rijad Konjhodzic, Nihad Fejzic, Sejla Goletic, Toni Eterovic, Adis Softic, Aida Kustura, Lana Salihefendic, Maja Ostojic, Maja Travar, Visnja Mrdjen, Nijaz Tihic, Sead Jazic, Sanjin Musa, Damir Marjanovic, Mirsada Hukic

**Affiliations:** 1 Veterinary Faculty of University of Sarajevo, Sarajevo, Bosnia and Herzegovina; 2 ALEA Genetic Center, Sarajevo, Bosnia and Herzegovina; 3 University Clinical Hospital of Mostar, Mostar, Bosnia and Herzegovina; 4 University Clinical Centre of the Republic of Srpska, Banja Luka, Bosnia and Herzegovina; 5 Faculty of Medicine, University of Banja Luka, Bosnia and Herzegovina; 6 University Clinical Center Tuzla, Tuzla, Bosnia and Herzegovina; 7 General Hospital “Abdulah Nakaš”, Sarajevo, Bosnia and Herzegovina; 8 Institute for Public Health of Federation of Bosnia and Herzegovina, Sarajevo, Bosnia and Herzegovina; 9 Center for Applied Bioanthropology, Institute for Anthropological Researches, Zagreb, Croatia and Faculty of Engineering and Natural Sciences, International Burch University, Sarajevo, Bosnia and Herzegovina; 10 The Academy of Science and Arts of Bosnia and Herzegovina, Sarajevo, Bosnia and Herzegovina

**Keywords:** Viral infections, COVID-19, SARS-CoV-2, whole genome sequencing, nanopore sequencing, phylogeny, Bosnia and Herzegovina

## Abstract

This is the first report of molecular and epidemiology findings from Bosnia and Herzegovina related to ongoing severe acute respiratory syndrome coronavirus 2 epidemic. Whole genome sequence of four samples from coronavirus disease 2019 (COVID-19) outbreaks was done in two laboratories in Bosnia and Herzegovina (Veterinary Faculty Sarajevo and Alea Genetic Center). All four BiH sequences cluster mainly with European ones (Italy, Austria, France, Sweden, Cyprus, and England). The constructed phylogenetic tree indicates possible multiple independent introduction events. The data presented contribute to a better understanding of COVID-19 in the current reemergence of the disease.

## INTRODUCTION

The first case of a new viral respiratory disease (coronavirus disease 2019 [COVID-19]) caused by a newly discovered virus (severe acute respiratory syndrome coronavirus 2 [SARS-CoV-2]) was confirmed in Bosnia and Herzegovina (BH) on March 5, 2020. In the period until June 20, a total of 3288 cases were confirmed, of which 168 with fatal outcome [1-2]. The elderly (>65), especially, are shown to be particularly sensitive to COVID-19 and suffer a higher mortality rate than the rest of the population [3]. On March 17, 2020, BH declared national emergency followed by the introduction of massive public health interventions to mitigate the outbreak, including mandatory self-isolation and quarantine on return to the country and enforced lockdowns. Despite having 13 different medical authorities and no state Ministry of Health due to complex governmental structure, it is generally agreed that BH authorities successfully implemented control measures, resulting in low morbidity and mortality [4]. Cease of restrictive measures started end-April 2020 but, with the increased number of COVID-19 cases in the first half of June, authorities are facing a new threat of an uncontrolled resurgence in COVID-19 transmission [5].

## MATERIALS AND METHODS

**FIGURE 1:**
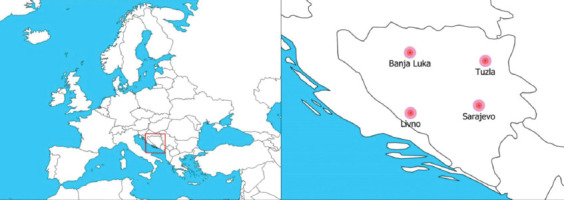
Samples were collected from four geographically representative different regions in BH (right): Livno, Banja Luka, Tuzla and Sarajevo.

The samples used in this study were nasopharyngeal swabs in viral transport medium from four patients, originating from Tuzla, Sarajevo, Livno, and Banja Luka, and real-time polymerase chain reaction (RT-PCR) COVID-19 confirmed on April 6, 8, 11, and 29, 2020, respectively (Figure 1).

Samples from Livno and Banja Luka were processed at the Laboratory of Veterinary Faculty Sarajevo (VFS) and samples from Tuzla and Sarajevo at the Alea Genetic Center, Sarajevo (AGC). A total of 140 μL of each sample was used for RNA extraction by QIAamp Viral RNA Mini Kit (Qiagen, Germany), and the product of extraction was later used for further analysis, while the rest of the samples were used for virus isolation on various cell lines at the VFS (data not shown here). The presence of SARS-CoV-2 in the samples was confirmed by real-time RT-PCR using HKU protocol [6], and the cycle threshold (Ct) values were 17.1; 21.3; 24.6; and 20.4 for Livno, Banja Luka, Tuzla and Sarajevo samples, respectively.

Whole genome sequencing (WGS) of Livno and Banja Luka samples was performed according to the ARTIC amplicon sequencing protocol for MinION for nCoV-2019, which uses two primer pools to generate the sequence, as described elsewhere [7]. The sequencing was performed on a MinION sequencer, using an R9.4.1 flow cell on which the samples, as well as a negative control, were pooled. MinKNOW software and the MinIT device were used for high accuracy real-time base calling during the run, which lasted 17 hours. Only the base-called FASTQ files with the Q score ≥7 were used for further analysis. The bioinformatic analysis was performed according to the nCoV-2019 novel coronavirus bioinformatics protocol [8]. The consensus sequence was mapped, for correction purposes, to the Wuhan reference genome (GenBank: MN908947) using Minimap2, and polished in Racon. WGS of Tuzla and Sarajevo samples was performed according to Ion AmpliSeq™ SARS-CoV-2 Research Panel Instructions for use on an Ion GeneStudio™ S5 Series System, as described previously [9], with minor modifications. Sequencing was performed on Ion GeneStudio™ S5 instrument using 520 chip. Raw data were analyzed using Torrent Suite Software 5.12.0 where the sequences were aligned to the Ion AmpliSeq SARS-CoV-2 reference genome. FASTA format of the obtained sequences was generated by the Iterative Refinement Meta-Assembler (IRMA) plugin. Sequences were additionally reviewed using BioEdit software.

To perform a phylogenetic analysis (PA) of BH sequences, a dataset of 120 whole genome sequences was obtained from GISAID (Supplementary material). These sequences were chosen based on their similarity with four BH sequences mentioned above. Namely, a set of 30 sequences from other countries, displaying the highest sequence identity with each BH sequence, were chosen. Sequence alignment and the construction of the phylogenetic tree were performed in MEGA X [10]. The phylogenetic tree was created using the maximum likelihood method, Hasegawa-Kishino-Yano substitution model, as well as 1000 bootstrap replicates.

## RESULTS

Four sequences were obtained from Livno, Banja Luka, Tuzla, and Sarajevo and named accordingly. All sequences were deposited in the Global Initiative on Sharing All Influenza Data (GISAID; https://www.gisaid.org/epiflu-applications/next-hcov-19-app/), with Accession IDs as follows: EPI_ISL_462753 (Livno sequence), EPI_ISL_462990 (Banja Luka sequence), EPI_ISL_463893 (Tuzla sequence), and EPI_ISL_467300 (Sarajevo sequence).

**FIGURE 2:**
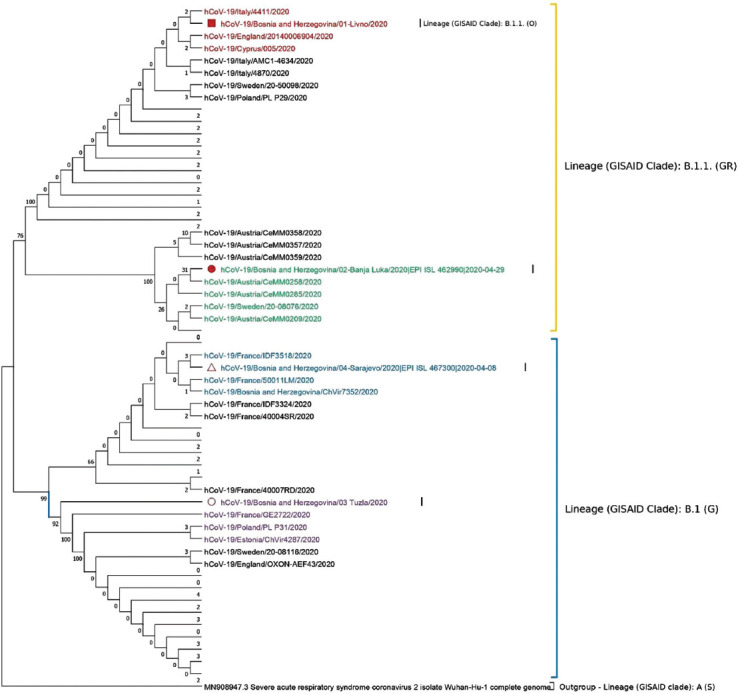
Phylogenetic tree of four SARS-CoV-2 genome sequences retrieved in this study, as well as sequences from different countries (*n* = 120 genome sequences). The phylogenetic tree was created using the Maximum Likelihood method, Hasegawa-Kishino-Yano substitution model as well as 1000 bootstrap replicates. Main clusters are highlighted in different colors. Certain branches have been collapsed and labels have been deleted for the ease of browsing.

The result of phylogenetic analysis is presented in Figure 2. Two main GISAID lineages [11] were highlighted with differently colored brackets and named accordingly: B.1.1. (GR) (yellow bracket) and B.1 (G) (blue bracket). Four BH sequences were marked with a different marker (red square, circles, and a triangle), and bootstrap values were shown at the level of nodes. Livno sequence (GISAID Accession ID: EPI_ISL_462753) is marked differently from other three BH sequences because its GISAID subclade (B.1.1. (O)) is different than the subclade it has been sorted in this phylogenetic tree (B.1.1. (GR)).

Position 614 in Spike protein, used to characterize the G clade [12], was shown to be G in BH isolates, assigning all four BH isolates to G clade together with European sequences (Italy, Austria, France, Sweden, Cyprus, and England). The constructed phylogenetic tree in Figure 2 indicates possible multiple independent introduction events as reflected by clustering of each single BH sequence in a separate cluster, highlighted with red (Livno, EPI_ISL_462753), green (Banja Luka, EPI_ISL_462990), blue (Sarajevo, EPI_ISL_467300), and purple (Tuzla, EPI_ISL_463893) (Figure 2.)

## DISCUSSION

We sequenced the whole genome out of four samples originating from Tuzla, Livno, Sarajevo, and Banja Luka municipalities, which are centers of four different BH geographical regions. All four BH sequences cluster mainly with European ones and the constructed phylogenetic tree indicates multiple independent introductions of COVID-19 in BH. Those findings correspond with other COVID-19 WGS studies [13,14] and confirm the importance of travel-associated disease ­introduction events for BH as well. The success of future containment/mitigation measures related to this type of disease introduction will be highly challenging for BH due to a significant proportion of its citizens living abroad and limited resources available to public health institutions. Hence, the WGS is a valuable approach for further investigation of the association level of various international movement modes (touristic, economic, religious, family visits, etc.) with transmission chains and associated risks.

Recently, a significant increase in the number of new cases (more than 390 in just 1 week due to imported cases as well as community transmission) emphasized a need to establish a more efficient scientific and expert system for further work on the genetic analysis of the SARS-CoV-2 virus, and to continue the investigation of the whole genetic COVID-19 pattern as well. Integration of phylogenetic (molecular) and epidemiological approaches in the assessment of human, animal, and environmental data will help with the identification of risk factors for disease spreading and optimize efficient and rational use of preventive and control measures.

The results gained from such approaches and studies may greatly assist in communicating the scientific base for the public health measures, thus improving the perception of the measures and the awareness of the general population.
